# Prognostic value of sarcopenia and inflammatory indices synergy in patients with esophageal squamous cell carcinoma undergoing chemoradiotherapy

**DOI:** 10.1186/s12885-024-12602-1

**Published:** 2024-07-18

**Authors:** Ling Xiao, Yudi Liu, Xue Zhang, Xinyu Nie, Hansong Bai, Jiahua Lyu, Tao Li

**Affiliations:** 1 School of Medicine, University of Electronic Science and Technology of China, Sichuan Cancer Hospital, Affiliated Cancer Hospital of University of Electronic Science and Technology of China, Chengdu, China; 2 Department of Radiation Oncology, Sichuan Clinical Research Center for Cancer, Sichuan Cancer Hospital & Institute, Sichuan Cancer Center, Affiliated Cancer Hospital of University of Electronic Science and Technology of China, Chengdu, China

**Keywords:** Esophageal squamous cell carcinoma, Chemoradiotherapy, Sarcopenia, Neutrophil-to-lymphocyte ratio

## Abstract

**Background and purpose:**

Sarcopenia has been demonstrated to be adversely correlated with the prognosis of various cancers. Our study aimed to estimate the prognostic value of sarcopenia in conjunction with inflammatory indices [neutrophil-to-lymphocyte ratio (NLR)] for evaluating the prognosis of patients with esophageal squamous cell carcinoma (ESCC) undergoing chemoradiotherapy.

**Materials and methods:**

This study retrospectively analyzed 255 patients with ESCC who received chemoradiotherapy from January 2012 to December 2018. Multivariate Cox regression analysis was employed to identify prognostic values of assessed factors following a novel prognostic scoring system (SMI-NLR), covering sarcopenia and NLR during different treatment courses.

**Results:**

Kaplan–Meier analysis revealed significantly greater overall survival (OS) rates in the nonsarcopenia group than in the sarcopenia group (*P* = 0.011). The low NLR group (< 4.84) demonstrated significantly higher OS rates than the high NLR group (≥ 4.84) (*P* < 0.001). The SMI-NLR prognostic model was established through multivariate analysis, revealing that Karnofsky performance status [hazard ratio (HR) = 0.285; 95% confidence interval (CI) = 0.117–0.699; *P* = 0.006], clinical staging (HR = 5.223; 95% CI = 1.879–14.514; *P* = 0.002), and preSMI-NLR (HR = 0.544; 95% CI = 0.330–0.898; *P* = 0.017) were independent factors affecting the prognosis of patients with ESCC. Nomograms were constructed based on these data providing more accurate 1-, 3-, and 5-year survival rates for patients with ESCC.

**Conclusion:**

Our study indicates the effectiveness of the combined sarcopenia and NLR prognostic model for the prognostic evaluation of patients with ESCC having undergone chemoradiotherapy.

**Supplementary Information:**

The online version contains supplementary material available at 10.1186/s12885-024-12602-1.

## Introduction

Esophageal malignancies have gained global attention due to their high incidence and mortality rates. Recent data indicate over 570,000 new cases annually [1]. Esophageal cancer is primarily categorized into esophageal squamous cell carcinoma (ESCC) and adenocarcinoma, with a significantly varying geographical distribution, and adenocarcinoma is predominant in Europe and America, whereas ESCC is more common in Asia [2–4]. Surgical resection remains the treatment of choice, but the condition is discovered at advanced stages in approximately 70% of patients, and chemoradiotherapy is the recommended standard of care for those unable or unwilling to undergo surgery [5–7]. Smoking, alcohol consumption, and poor dietary habits possess the greatest risks for developing esophageal cancer making effective cessation and early screening and diagnosis of paramount importance [4, 8, 9]. The overall prognosis remains poor despite the elaboration of new strategies, including targeted and immunotherapies, offering more options to patients [10, 11].

Currently, tumor staging serves as an essential measure for guiding clinical treatment and evaluating prognoses in patients with esophageal cancer; however, significant disparities remain within the same clinical stages in the latter [12]. Therefore, the development of effective screening tools determined high-risk individuals and conducted tailored treatment demonstrated significant clinical value. Recent studies have revealed that easily measurable blood biomarkers, such as neutrophil-to-lymphocyte ratio (NLR), platelet-to-lymphocyte ratio (PNI), and systemic immune-inflammation index, can reflect systemic inflammation levels along with the efficacy of performed treatment and survival prognosis in patients with cancer [13–15].

Sarcopenia, as outlined by the European Working Group on Sarcopenia in Older People and the Asian Working Group on Sarcopenia, is a syndrome exhibiting progressively reduced and generalized loss of skeletal muscle mass and strength associated with physical disability, deteriorated quality of life, and higher mortality risks [16–18]. Recent research indicates that systemic inflammation may be an integral part of the onset of sarcopenia and that the prognostic properties of combined evaluation of systemic inflammation and sarcopenia reveal more stability and effectiveness than single-factor [19–21]. The interaction of the factors in pre-treatment sarcopenia and systemic inflammation and their potential effect on the outcomes in patients with esophageal cancer remains unclear.

However, the synergistic effect of sarcopenia and systemic inflammation on the prognosis of patients with esophageal cancer remains unclear. Hence, this study focuses on analyzing the interplay between sarcopenia and the NLR in patients with esophageal cancer subjected to chemoradiotherapy and its effect on patient prognosis. We aim to construct a novel prognostic scoring model based on these biomarkers to forecast more precisely long-term survival outcomes for patients receiving chemoradiotherapy and provide more targeted and effective treatment plans. Additionally, we analyzed the changes in skeletal muscle index (SMI) and NLR levels before and after therapy and their influence on prognoses, further validating the accuracy of prognostic prediction with these factors through subgroup analysis.

## Materials and methods

### Study population

This study retrospectively evaluated 255 patients with ESCC who received chemoradiotherapy at Sichuan Cancer Hospital from January 2012 to December 2018. The following inclusion criteria were applied: (1) histologically confirmed ESCC; (2) ESCC that was either unresectable or the patient was unwilling to undergo surgery; (3) a Karnofsky performance status (KPS) score of ≥ 70; (4) a radiation dose of ≥ 40 Gy; (5) availability of complete blood count from one week before treatment initiation; (6) no evidence of distant metastases; (7) TNM staging based on the 7th edition of the American Joint Committee on Cancer staging system.

### Treatment

All the patients underwent radiotherapy, with spiral computed tomography (CT) scans used for tumor target delineation and visualizing adjacent nonaffected organs. Three-dimensional conformal radiotherapy (3D-CRT) or intensity-modulated radiotherapy (IMRT), with PTV doses of 50–72 Gy over 25–36 fractions, were performed across 5–7 weeks. Constraints were set with V20 of ≤ 25% for both lungs, V30 of ≤ 40% for the heart, V40 of ≤ 30%, and a maximum spinal cord dose of ≤ 45.0 Gy. Some patients received 1–6 cycles of platinum-based monotherapy or combination chemotherapy, while older patients were subjected to oral tegafur treatment, and those intolerant to first-line chemotherapy were given raltitrexed.

Blood biomarker calculation methods: Pre-treatment and post-treatment serum albumin levels, lymphocyte counts, and neutrophil counts were collected. According to previous reports, PNI was defined as 10 × serum albumin level (g/dL) + 0.005 × lymphocyte count (mm^3^); NLR was defined as the neutrophil count divided by the lymphocyte count [15].

### Body composition calculation

Patient CT images were obtained from the PACS system of the Sichuan Cancer Hospital Imaging Department. Two trained radiologists, blinded to the outcomes, delineated the skeletal muscle area (SMA) at the L3 level using semiautomated SliceOmatic software (version 5.0; Tomovision, Montreal, QC, Canada), based on attenuation thresholds of -29 to + 150 Hounsfield Units, that covered psoas, quadratus lumborum, erector spinae, transversus abdominis, internal oblique, external oblique, and rectus abdominis muscles. The SMI was calculated as SMA (cm2)/height2 (m2) [22], with the diagnostic thresholds for sarcopenia set at 40.8 cm2/m2 for men and 34.9 cm2/m2 for women following the research within Asian populations [18].

### Outcome measures and follow-up

Follow-up was conducted every 3 months in the first year, every 6 months in the following 2 years, and annually thereafter. Routine evaluations included physical examinations, blood tests, ultrasonography, tumor marker assays, chest CT imaging, and esophagography with barium meal. All patients were followed up through outpatient visits and telephone interviews.

### Statistical methods

Statistical Package for the RStudio version 4.3.1 (R Foundation for Statistical Computing) were used for data analysis and visualization. Chi-square or Fisher’s exact test was applied for categorical variables and Student’s *t*-test for continuous variables. The optimal stratification of continuous covariates for different treatment stages, such as NLR, PNI, and SMI-NLR cut-off values, was performed with log-rank tests [23, 24]. Patients were allocated to low and high groups according to the latter. The Kaplan–Meier method, with differences between curves analyzed using the log-rank test, was used to plot Survival curves. Uni- and multivariate analyses were performed with Cox proportional hazards models and a significance level was established at α of 0.05 and the statistical significance at a *p*-value of < 0.05.

## Results

### Patient characteristics

This study enrolled 255 patients with ESCC meeting the inclusion criteria. The median age of the participants was 64 years (range: 34–87 years), while the gender distribution was reflected in 200 (78.5%) men and 55 (21.5%) women. Of them, 147 (57.6%) and 108 (42.4%) patients reported with and without smoking habit. Alcohol consumption was reported in 140 (54.9%) patients, while 115 (45.1%) were non-drinkers. Tumors of < 5 cm in size were observed in 102 cases (40.0%), and those of ≥ 5 cm were observed in 153 (60.0%) patients. Stages I–II of the disease were noted in 16 (6.3%) cases, and stages III–IV were detected in 239 (96.7%) cases. Additionally, 39 (15.3%) patients received no chemotherapy, whereas 216 (84.7%) patients did (Table 1).

**Table 1:** The distribution of background variables stratified by survival status

**Characteristics**	**Total, n (%)**	**Live(n)**	**Dead(n)**	***P* value**
OS, median (IQR)		44 (37.118, 60.843)	12.3 (7.87, 22.7)	< 0.001
Gender, n (%)				0.851
Male	200 (78.5%)	70 (27.5%)	130 (51%)	
Female	55 (21.5%)	20 (7.8%)	35 (13.7%)	
Age, mean ± sd		63.422 ± 7.8768	64.085 ± 10.121	0.591
KPS, n (%)				0.028
70	6 (2.4%)	0 (0%)	6 (2.4%)	
80	107 (42.0%)	31 (12.2%)	76 (29.8%)	
90	140 (54.9%)	59 (23.1%)	81 (31.8%)	
100	2 (0.8%)	0 (0%)	2 (0.8%)	
Smoking history, n (%)				0.445
No	108 (42.4%)	41 (16.1%)	67 (26.3%)	
Yes	147 (57.6%)	49 (19.2%)	98 (38.4%)	
Alcohol history, n (%)				0.091
No	115 (45.1%)	47 (18.4%)	68 (26.7%)	
Yes	140 (54.9%)	43 (16.9%)	97 (38%)	
Tumorlocation, n (%)				0.428
Cervical	8 (3.2%)	5 (2%)	3 (1.2%)	
Upper thoracic	53 (20.7%)	21 (8.2%)	32 (12.5%)	
Middle thoracic	96 (37.6%)	32 (12.5%)	64 (25.1%)	
Lower thoracic	86 (33.8%)	29 (11.4%)	57 (22.4%)	
Abdominal	12 (4.7%)	3 (1.2%)	9 (3.5%)	
Tumor length(cm), n (%)				0.061
< 5	102 (40%)	43 (16.9%)	59 (23.1%)	
≥ 5	153 (60%)	47 (18.4%)	106 (41.6%)	
Clinicalstage, n (%)				< 0.001
I-II	16 (6.3%)	12 (4.7%)	4 (1.6%)	
III-IV	239 (96.7%)	78 (30.6%)	161 (63.1%)	
BMI, mean ± sd		22.066 ± 2.8334	21.591 ± 2.8761	0.206
RT dose, median (IQR)		66 (60, 66)	66 (64, 66)	0.048
Chemotherapy, n (%)				0.170
No	39 (15.3%)	10 (3.9%)	29 (11.4%)	
Yes	216 (84.7%)	80 (31.4%)	136 (53.3%)	
SMA, mean ± sd		120.84 ± 23.689	117.85 ± 22.16	0.315
SMI, mean ± sd		46.55 ± 8.1754	44.501 ± 7.7398	0.049
PNI, median (IQR)		49.825 (46.912, 52.237)	48.5 (45, 51.65)	0.073
NLR, median (IQR)		2.55 (1.94, 3.5925)	2.89 (1.94, 4.49)	0.064
SMI-PNI, n (%)				0.016
< 94.13	128 (50.2%)	36 (14.1%)	92 (36.1%)	
≥ 94.13	127 (49.8%)	54 (21.2%)	73 (28.6%)	
SMI-NLR, n (%)				0.003
< 11.04	65 (25.5%)	13 (5.1%)	52 (20.4%)	
≥ 11.04	190 (74.5%)	77 (30.2%)	113 (44.3%)	

*KPS* Karnofsky performance score, *BMI* Body Mass Index, *SMA* Skeletal muscle area, *SMI* Skeletal muscle index, *PNI* Prognostic nutritional index, *NLR* Neutrophil-to-lymphocyte ratio, *RT* radiotherapy

### The relationship between patient prognosis and sarcopenia

**Fig. 1:**
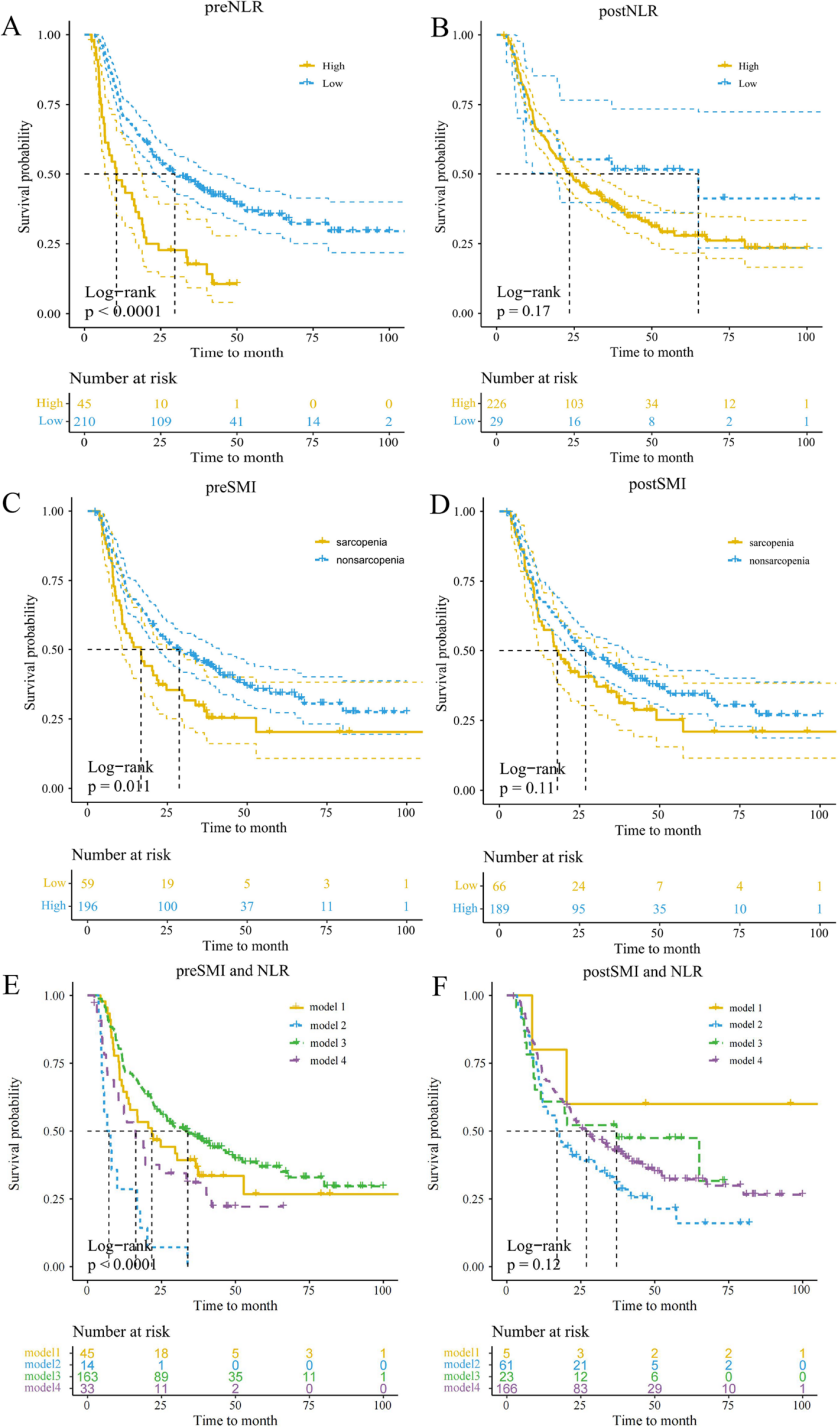
The Kaplan–Meier survival curves of SMI、NLR in the OS of overall. preSMI, pre-treatment skeletal muscle index; preNLR, pre-treatment neutrophil-to-lymphocyte ratio, postSMI, post-treatment skeletal muscle index; postNLR, post-treatment neutrophil-to-lymphocyte ratio

In a study of 255 patients, 1-, 3-, and 5-year overall survival (OS) rates were 68.9%, 41.8%, and 30.9%, respectively. Patients with a pre-treatment NLR (preNLR) < 4.84 showed higher OS rates of 73.1%, 46.3%, and 34.7%, compared to those with NLR ≥ 4.84, who had OS rates of 50.0%, 21.4%, and 15.3%. Kaplan–Meier analysis revealed significantly higher OS in the former group (*p* < 0.001, Fig. 1A and B). Additionally, non-sarcopenic patients had 1-, 3-, and 5-year OS rates of 72.3%, 45.4%, and 34.1%, respectively, surpassing the 57.6%, 37.3%, and 20.3% rates observed in sarcopenic patients. Kaplan–Meier analysis significantly higher OS rates in the former than in the latter (*p* = 0.011, Fig. 1C and D).

### Skeletal muscle mass index and sarcopenia incidence

**Fig. 2:**
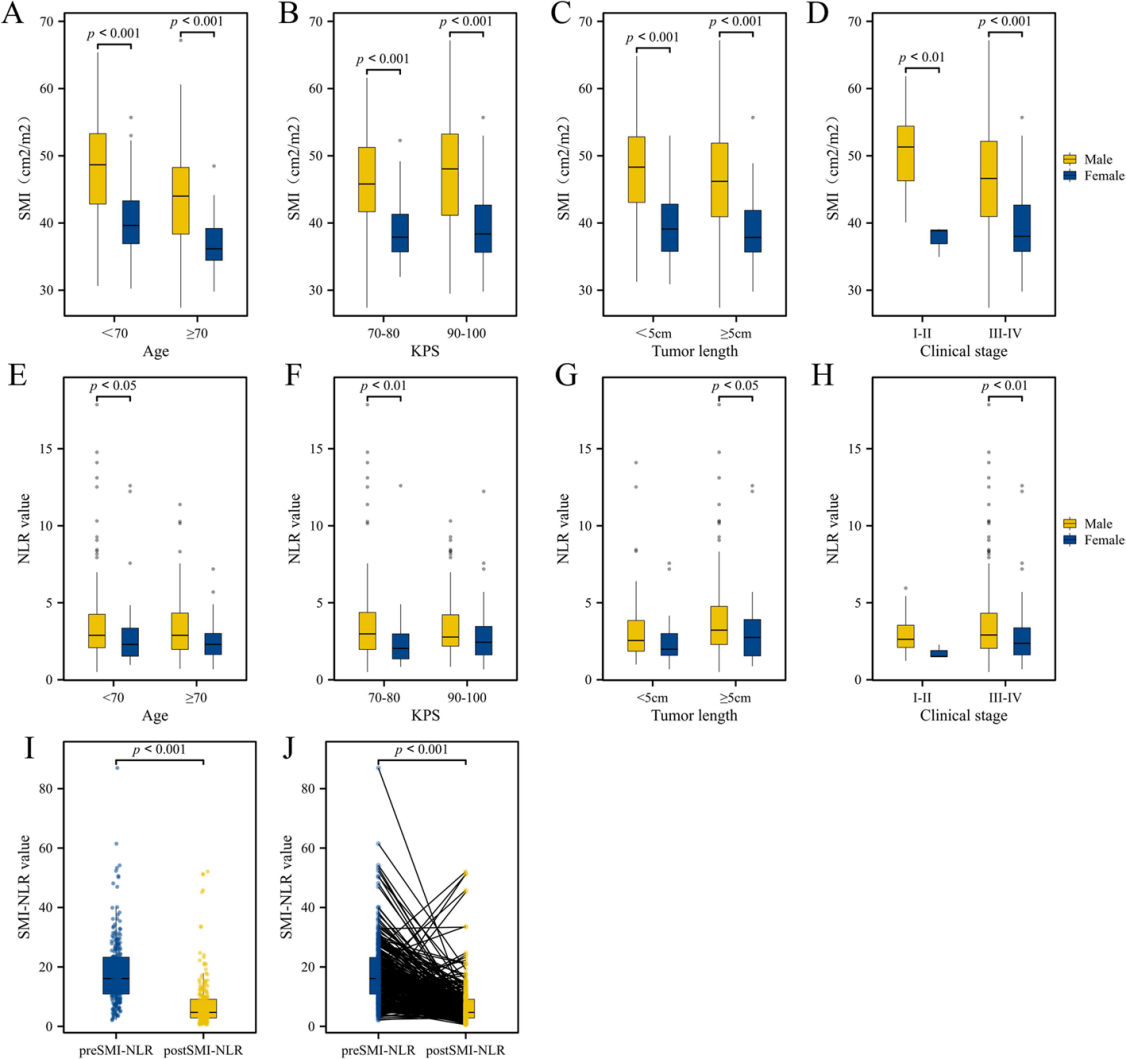
The distribution of SMI in different groups. KPS, Karnofsky performance score; BMI, Body Mass Index; SMI, skeletal muscle index; NLR, neutrophil-to-lymphocyte ratio; PreSMI-NLR, pre-treatment skeletal muscle index combined with pre-treatment neutrophil-to-lymphocyte ratio; postSMI-NLR, post-treatment skeletal muscle index combined with post-treatment neutrophil-to-lymphocyte ratio

Pre-treatment SMI (preSMI) and preNLR levels were analyzed in gender groups, and the associations with age, KPS score, tumor length, and clinical stage were revealed, all demonstrating statistical significance (*p* < 0.05, Fig. 2A-H). Sarcopenia was present in 59 (23.1%) patients before treatment, including 47 men (23.5%) and 12 women (21.8%). Sarcopenia was found in 66 (25.9%) patients, including 51 men (25.5%) and 15 women (27.3%), after the therapy (Table 2).

**Table 2:** The distribution of background variables stratified by survival status

**Characteristics**	**Male (*n* = 200)**	**Female (*n* = 55)**	***P* value**
Gender, n (%)	70 (27.5%)	20 (7.8%)	
Male	130 (51%)	35 (13.7%)	
Female	22.935 (10.035, 40.532)	22.03 (11.65, 42.6)	0.767
Age, mean ± sd	63.23 ± 9.4925	66.109 ± 8.6681	0.044
KPS, n (%)			0.505
70	6 (2.4%)	0 (0%)	
80	84 (32.9%)	23 (9%)	
90	108 (42.4%)	32 (12.5%)	
100	2 (0.8%)	0 (0%)	
Smoking history, n (%)			< 0.001
No	55 (21.6%)	53 (20.8%)	
Yes	145 (56.9%)	2 (0.8%)	
Alcohol history, n (%)			< 0.001
No	64 (25.1%)	51 (20%)	
Yes	136 (53.3%)	4 (1.6%)	
Tumorlocation, n (%)			0.440
Cervical	6 (2.4%)	2 (0.8%)	
Upper thoracic	40 (15.7%)	13 (5.1%)	
Middle thoracic	74 (29%)	22 (8.6%)	
Lower thoracic	68 (26.7%)	18 (7.1%)	
Abdominal	12 (4.7%)	0 (0%)	
Tumor length(cm), n (%)			0.534
< 5	78 (30.6%)	24 (9.4%)	
≥ 5	122 (47.8%)	31 (12.2%)	
Clinicalstage, n (%)			1.000
III-IV	187 (73.3%)	52 (20.4%)	
I-II	13 (5.1%)	3 (1.2%)	
BMI, mean ± sd	21.295 (19.802, 23.258)	22.03 (20.26, 24.68)	0.190
RT dose, median (IQR)	66 (61.85, 66)	66 (64.35, 66)	0.413
Chemotherapy, n (%)			0.502
No	171 (67.1%)	45 (17.6%)	
Yes	29 (11.4%)	10 (3.9%)	
PNI, median (IQR)	48.85 (45.637, 51.713)	49.85 (46.45, 53.2)	0.102
NLR, median (IQR)	2.89 (2.04, 4.265)	2.3 (1.555, 3.35)	0.004
SMI, n (%)			0.793
Nonsarcopenia	153 (60%)	43 (16.9%)	
Sarcopenia	47 (18.4%)	12 (4.7%)	

*KPS* Karnofsky performance score, *BMI* Body Mass Index, *SMA* Skeletal muscle area, *SMI* Skeletal muscle index, *PNI* Prognostic nutritional index, *NLR* Neutrophil-to-lymphocyte ratio, *RT* radiotherapy

### Correlation between sarcopenia and inflammation

Uni- and multivariate logistic regression analyses were performed to investigate the correlation between systemic inflammation levels and SMI. The former identified clinical stage and preNLR as predictors for preSMI. Multivariate analysis revealed that clinical stage (hazard ratio [HR] = 0.494, 95% confidence interval [CI] = 0.290–0.844; *p* = 0.010) and preNLR (HR = 1.099; 95% CI = 1.039–1.162; *p* = 0.001) were the essential predictors for preSMI (Table 3). Considering the significant role of inflammatory components in sarcopenia, combined with the aforementioned analysis, we have hypothesized that SMI conjugated with NLR is a potentially effective tool for evaluating the prognoses in patients undergoing chemoradiotherapy. We combined SMI and NLR levels to create four subgroups to further differentiate patients according to distinct outcomes. Patients with sarcopenia and preNLR of ≥ 4.84 demonstrated the poorest survival rates, while those without the former and preNLR of < 4.84 exhibited the highest survival rates (*p* = 0.004). However, the survival rates for the patients from the former group were similar to those without sarcopenia but with preNLR of ≥ 4.84 (*p* > 0.05, Fig. 1E and F).

**Table 3:** Univariate and multivariate Cox regression analyses of factors associated with Sarcopenia

**Characteristics**	**Total(N)**	**Univariate analysis**		**Multivariate analysis**	
		**Hazard ratio (95% CI)**	***P* value**	**Hazard ratio (95% CI)**	***P* value**
Gender, n (%)	255				
Male	200	Reference			
Female	55	1.041 (0.741—1.461)	0.818		
Age, mean ± sd	255	0.998 (0.980—1.016)	0.827		
KPS, n (%)	255				
70	6	Reference			
80	107	2.546 (0.354—18.322)	0.353		
90	140	2.614 (0.364—18.752)	0.339		
100	2	7.500 (0.678—82.996)	0.100		
Smoking history, n (%)	255				
No	108	Reference			
Yes	147	0.977 (0.736—1.297)	0.872		
Alcohol history, n (%)	255				
No	115	Reference			
Yes	140	1.168 (0.880—1.550)	0.282		
Tumorlocation, n (%)	255				
Cervical	8	Reference			
Upper thoracic	53	1.203 (0.538—2.690)	0.653		
Middle thoracic	96	0.974 (0.447—2.123)	0.947		
Lower thoracic	86	0.785 (0.357—1.730)	0.549		
Abdominal	12	0.771 (0.286—2.082)	0.608		
Tumor length(cm), n (%)	255				
< 5	102	Reference			
≥ 5	153	1.221 (0.916—1.628)	0.174		
Clinicalstage, n (%)	255				
III-IV	239	Reference		Reference	
I-II	16	0.486 (0.285—0.829)	0.008	0.494 (0.290—0.844)	0.010
BMI, mean ± sd	255	1.010 (0.959—1.064)	0.706		
RT dose, median (IQR)	254	0.979 (0.956—1.003)	0.089	0.975 (0.952—0.999)	0.042
Chemotherapy, n (%)	255				
No	216	Reference			
Yes	39	1.315 (0.846—2.044)	0.224		
PNI, median (IQR)	255	0.980 (0.956—1.004)	0.107		
NLR, median (IQR)	255	1.092 (1.033—1.155)	0.002	1.099 (1.039—1.162)	0.001

*KPS* Karnofsky performance score, *BMI* Body Mass Index, *SMA* Skeletal muscle area, *SMI* Skeletal muscle index, *PNI* Prognostic nutritional index, *NLR* Neutrophil-to-lymphocyte ratio, *RT* Radiotherapy, *HR* Hazard ratio, *CI* Confidence interval

### Establishment of a prognostic model based on sarcopenia and NLR

**Fig. 3:**
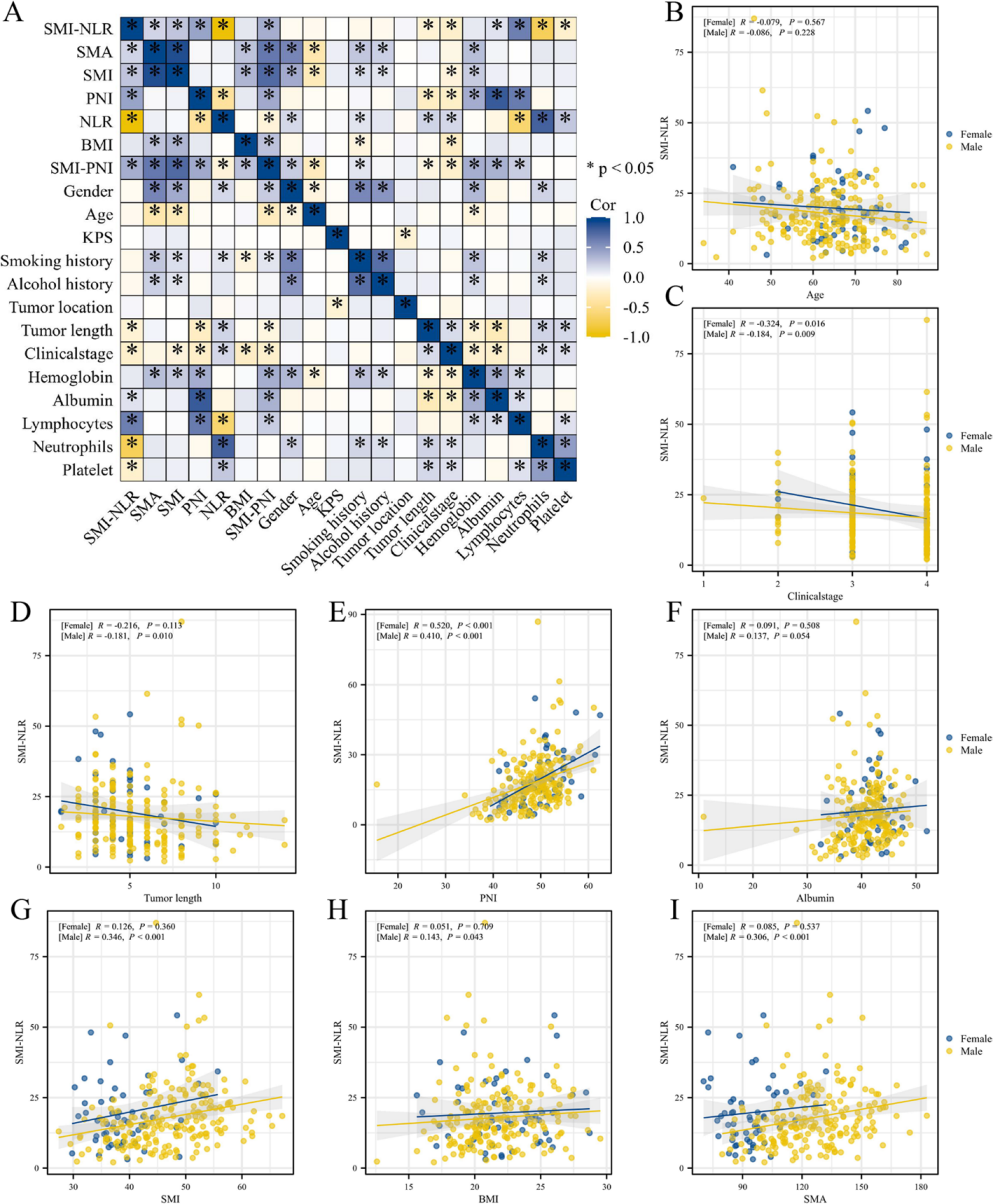
The Person analysis of SMI-NLR and related-factors. KPS, Karnofsky performance score; BMI, Body Mass Index; SMI, skeletal muscle index; NLR, neutrophil-to-lymphocyte ratio; SMI-NLR,skeletal muscle index combined with neutrophil-to-lymphocyte ratio

Optimal stratification was implemented to determine the best cut-off values for continuous covariates resulting in a pre-treatment SMI-NLR (preSMI-NLR) threshold of 11.04 and a post-treatment SMI-NLR (postSMI-NLR) of 3.53. The preSMI-NLR for the whole sample was 18.27 ± 11.20, and the postSMI-NLR composed 8.71 ± 24.6, showing a downward trend (Fig. 2I and J). Spearman’s analysis was performed on various clinical parameters associated with preSMI-NLR. Significant correlations were obtained for body mass index (BMI), SMA, SMI, PNI, NLR, tumor length, and clinical stage (*p* < 0.05, Fig. 3).

**Fig. 4:**
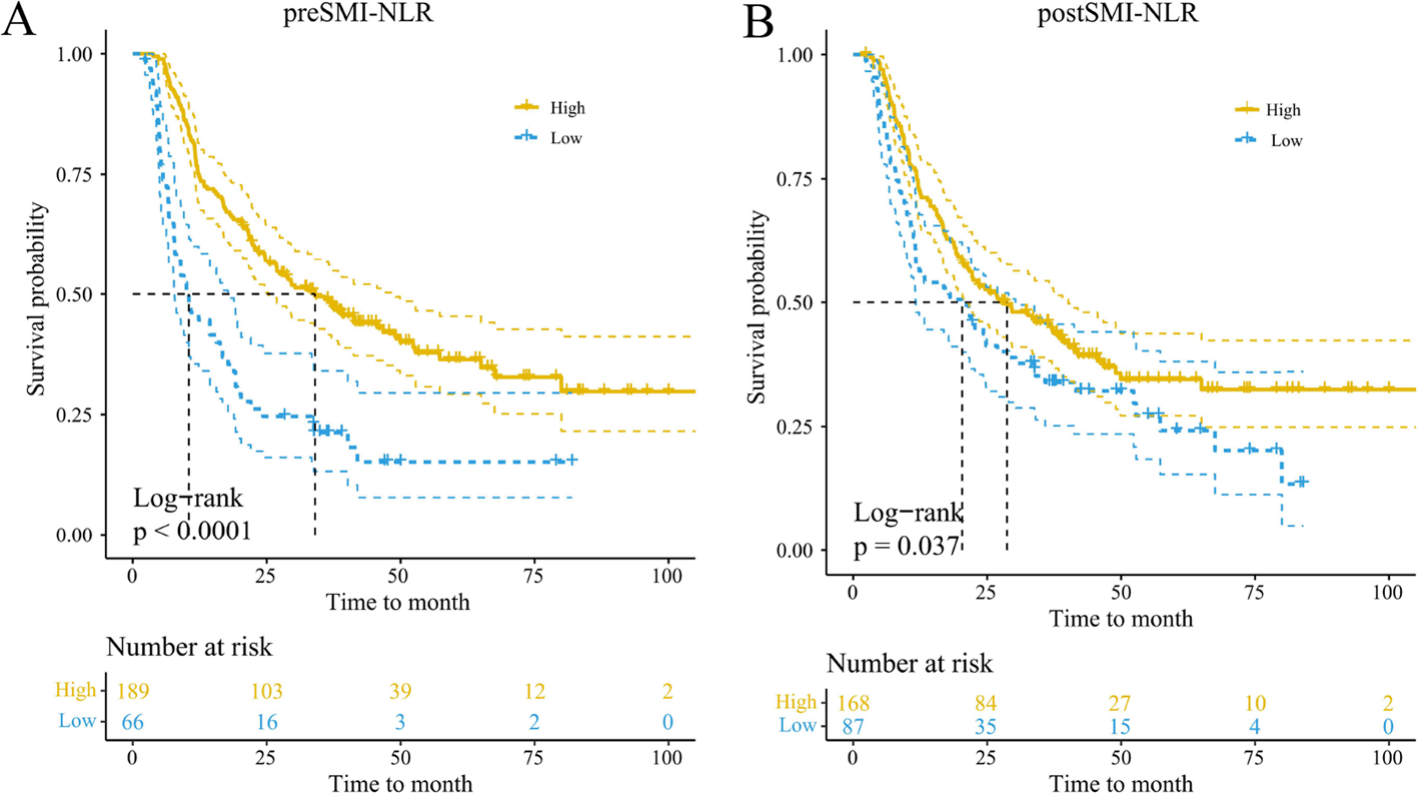
The Kaplan–Meier survival curves of SMI-NLR in the OS of overall. PreSMI-NLR, pre-treatment skeletal muscle index combined with pre-treatment neutrophil-to-lymphocyte ratio; postSMI-NLR, post-treatment skeletal muscle index combined with post-treatment neutrophil-to-lymphocyte ratio

Kaplan–Meier analysis revealed that the high preSMI-NLR group demonstrated significantly higher OS rates than the low preSMI-NLR (*p* < 0.001, Fig. 4). The univariate analysis reflected the link between KSP, tumor length, clinical stage, BMI, chemotherapy, SMI, PNI, NLR, and preSMI-NLR and the prognosis of patients with ESCC (*p* < 0.05). Multivariate analysis revealed that KSP (HR = 0.285; 95% CI = 0.117–0.699; *p* = 0.006), clinical stage (HR = 5.223; 95% CI = 1.879–14.514; *p* = 0.002), and preSMI-NLR (HR = 0.544; 95% CI = 0.330–0.898; *p* = 0.017) were independent factors that affected the prognosis of patients with ESCC (Table 4).

**Table 4:** Univariate and multivariate Cox regression analyses of factors associated with overall survival

**Characteristics**	**Total(N)**	**Univariate analysis**		**Multivariate analysis**	
		**Hazard ratio (95% CI)**	***P* value**	**Hazard ratio (95% CI)**	***P* value**
Gender, n (%)	255				
Male	200	Reference			
Female	55	0.964 (0.664—1.401)	0.849		
Age, mean ± sd	255	1.012 (0.993—1.030)	0.217		
KPS, n (%)	255				
70	6	Reference		Reference	
80	107	0.483 (0.210—1.110)	0.086	0.370 (0.152—0.900)	0.028
90	140	0.380 (0.166—0.872)	0.022	0.285 (0.117—0.699)	0.006
100	2	0.966 (0.195—4.795)	0.966	0.678 (0.128—3.585)	0.647
Smoking history, n (%)	255				
No	108	Reference			
Yes	147	1.161 (0.851—1.585)	0.346		
Alcohol history, n (%)	255				
No	115	Reference		Reference	
Yes	140	1.335 (0.979—1.821)	0.068	1.371 (0.991—1.896)	0.057
Tumorlocation, n (%)	255				
Cervical	8	Reference			
Upper thoracic	53	2.242 (0.686—7.327)	0.181		
Middle thoracic	96	2.234 (0.701—7.118)	0.174		
Lower thoracic	86	2.144 (0.670—6.860)	0.199		
Abdominal	12	2.300 (0.622—8.505)	0.212		
Tumor length(cm), n (%)	255				
< 5	102	Reference		Reference	
≥ 5	153	1.511 (1.098—2.078)	0.011	1.287 (0.924—1.792)	0.136
Clinicalstage, n (%)	255				
I-II	16	Reference		Reference	
III-IV	239	4.866 (1.800—13.153)	0.002	5.223 (1.879—14.514)	0.002
BMI, mean ± sd	255	0.932 (0.880—0.987)	0.015	0.952 (0.895—1.014)	0.127
RT dose, median (IQR)	254	1.012 (0.983—1.041)	0.430		
Chemotherapy, n (%)	255				
No	39	Reference		Reference	
Yes	216	0.596 (0.398—0.892)	0.012	0.643 (0.413—1.002)	0.051
pSMI	255	0.977 (0.959—0.996)	0.019	1.014 (0.980—1.048)	0.430
pPNI	255	0.969 (0.946—0.993)	0.011	0.992 (0.961—1.024)	0.617
pNLR	255	1.095 (1.040—1.154)	< 0.001	1.010 (0.932—1.094)	0.813
PreSMI-PNI	255				
< 94.13	128	Reference		Reference	
≥ 94.13	127	0.575 (0.423—0.784)	< 0.001	0.681 (0.401—1.155)	0.154
PreSMI-NLR	255				
< 11.04	65	Reference		Reference	
≥ 11.04	190	0.414 (0.297—0.577)	< 0.001	0.544 (0.330—0.898)	0.017

*KPS* Karnofsky performance score, *BMI* Body Mass Index, *SMA* Skeletal muscle area, *SMI* Skeletal muscle index, *PNI* Prognostic nutritional index, *NLR* Neutrophil-to-lymphocyte ratio, *RT* Radiotherapy, *HR* Hazard ratio, *CI* Confidence interval

### Construction of a risk-scoring prognostic model

**Fig. 5:**
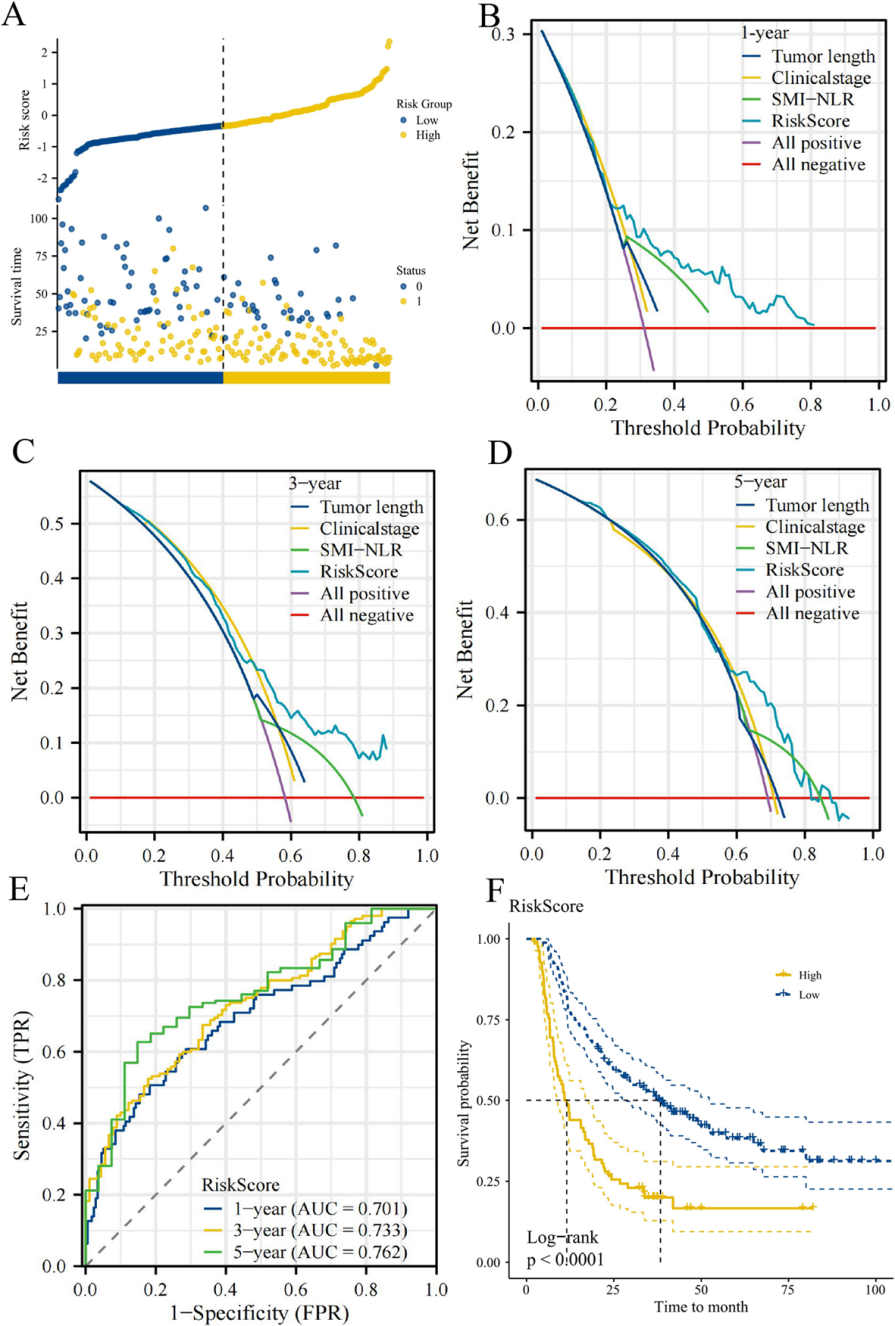
The prognostic risk score model of ECSS based on SMI-NLR score. AUC, area under curve; SMI-NLR,skeletal muscle index combined with neutrophil-to-lymphocyte ratio

**Fig. 6:**
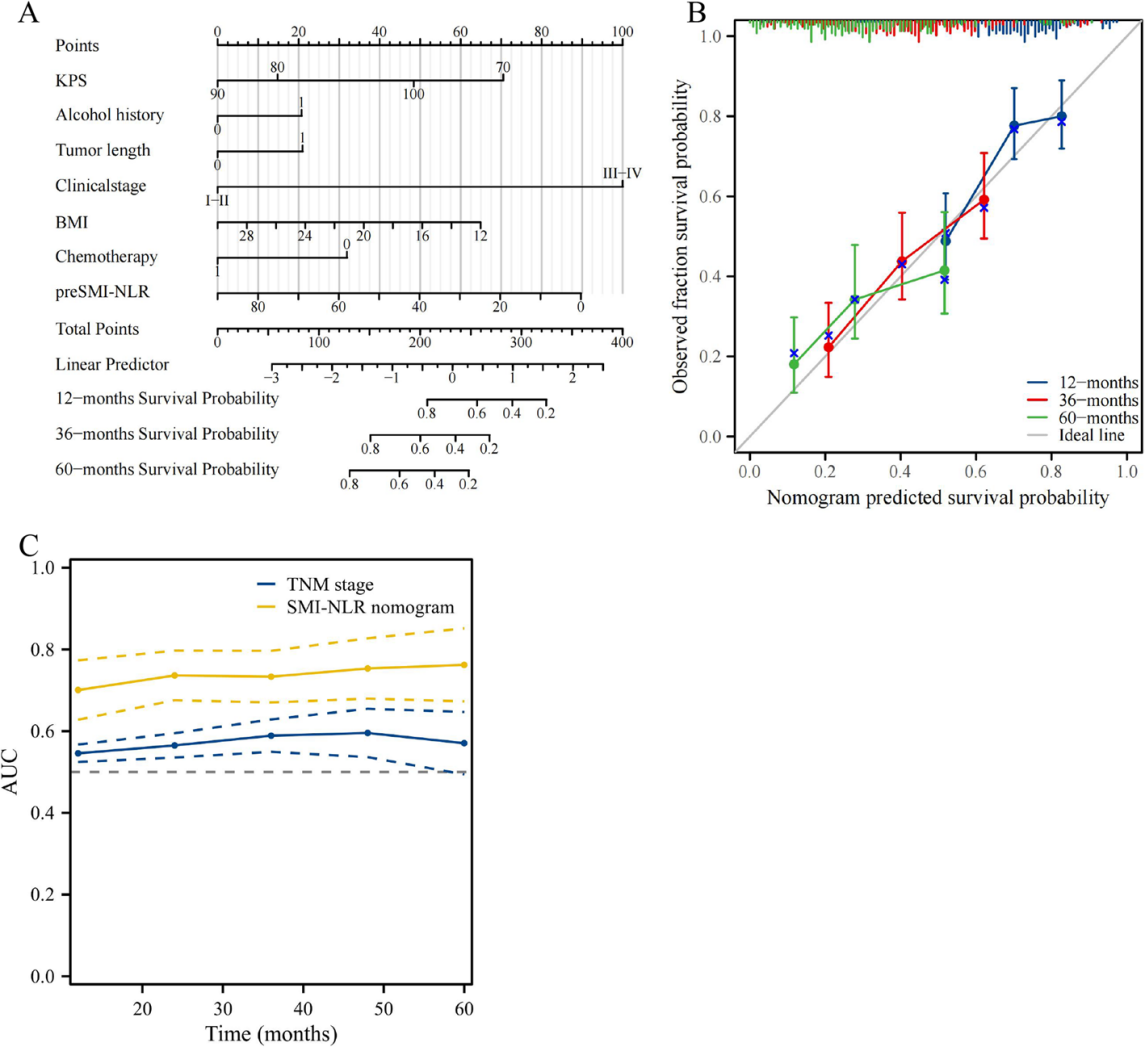
The Nomogram constructed of ECSS based on SMI-NLR score. KPS, Karnofsky performance score; BMI, Body Mass Index; preSMI-NLR, pre-treatment skeletal muscle index combined with pre-treatment neutrophil-to-lymphocyte ratio

This study revealed significant non-conformities in the prognoses within the same TNM stage while TNM staging is essential in the treatment and prognostic assessment in patients with cancer. We based our risk scoring on a multivariate Cox regression hazard model to provide a quantitative method for better outcome predictions. The risk score was designed to reveal the prognostic outcomes of patients. Heatmaps of prognosis, survival curves, and time-dependent survival curves for different risk scores significantly differed (*p* < 0.05). Results revealed that individuals with higher risk scores demonstrated lower survival rates than those with lower risk. The prognostic receiver operating characteristic curves for 1-, 3-, and 5-year areas under the curve were 70.0, 73.6, and 75.5, respectively (Fig. 5A-F). Furthermore, we designed a nomogram incorporating independent prognostic factors to precisely predict ESCC patient outcomes following radiotherapy and chemotherapy. This nomogram, validated by its superior predictive accuracy and well-performing calibration plots compared to the ideal model, effectively complements the TNM staging system (Fig. 6A-C).

## Discussion

Sarcopenia, a term first introduced by Rosenberg et al. [25] is a pathological condition that progressively develops with age, featuring a gradual decrease in overall muscle mass and strength leading to adverse events, such as falls and disability. Traditionally, sarcopenia has been considered a common phenomenon in the older adult age group. However, an increasing body of research has revealed a high incidence of sarcopenia in patients with cancer affecting not only their quality of life but also provoking adverse reactions to anti-cancer treatments and worsening prognoses [26, 27]. The skeletal muscle area at the third lumbar vertebra outlined in CT scans is the core part of SMI, which has become the “gold standard” for assessing muscle mass. SMI provides more information on body composition and can evaluate more accurately the nutritional status of patients.

Current studies reveal that sarcopenia significantly affects the survival rate of patients with head and neck, esophageal, lung, and gastrointestinal malignant tumors [20, 28–31]. Consequently, the definition of SMI varies considerably following ethnic and disease-specific characteristics. A study based on a European population defined the condition as that with SMI of < 52.4 cm^2/m^2 for men and SMI < 38.5 cm^2/m^2 for women. This study enrolled 97 patients with locally advanced esophageal cancer and revealed that the sarcopenia prevalence of 44% was associated with the increased overall mortality rate, with sarcopenia being an important prognostic risk factor (HR = 32.1 [24.4–34.0], *p* = 0.008) [31]. Similarly, Qian et al. used the aforementioned definition of sarcopenia and detected a prevalence rate of 79.8% in Asian patients with ESCC undergoing radiotherapy or CRT, which was associated with poorer PFS and OS rates. Sarcopenia, albumin levels, and tumor staging were determined as essential factors predicting survival [32].

The predictive role of sarcopenia is consistent with our study results; however, considering the differences in population ethnicity and disease types, our study adopted Asian population SMI cut-off values of 40.8 cm^2/m^2 for male patients and 34.9 cm^2/m^2 for female [18]. Our analysis investigated changes in SMI levels during different treatment processes, with sarcopenia occurring pre-treatment in 59 (23.1%) individuals and post-treatment in 66 (25.9%) individuals showing significant differences through paired analysis (*p* < 0.05). Furthermore, our study revealed that lower KPS levels and advanced clinical stages were significantly associated with decreased OS rates.

Multiple studies have indicated the pivotal role of chronic inflammation in the onset and progression of esophageal cancer [33, 34]. NLR is a biomarker reflecting systemic inflammatory status, and it has attracted attention in recent years for its application in evaluating prognoses for patients with cancer [35–37]. Neutrophils can secrete immunosuppressive mediators and angiogenic factors, forming tumor microenvironment and thereby promoting tumor progression [38, 39]. Conversely, circulating lymphocytes may inhibit tumor proliferation, invasion, and metastasis by enhancing tumor immune surveillance [40]. Our study revealed that patients with a low NLR had significantly higher survival rates than those with a high NLR. Nevertheless, postNLR failed to yield analogous outcomes. This discrepancy could stem from the inability of postNLR to fulfill its predictive function accurately, owing to myelosuppression, including neutropenia, and the employment of granulocyte colony-stimulating factors following radiotherapy and chemotherapy, which lead to significant variances in blood markers. Moreover, systemic inflammation levels were somewhat associated with the presence of cancer-related sarcopenia. Patients with esophageal cancer, who are prone to malnutrition due to prolonged inadequate food intake, advanced tumor staging, persistent high-inflammatory states, and intensive anti-tumor treatments, may activate the ubiquitin–proteasome pathway. This process can cause elevated levels of inflammatory cytokines, such as interleukin-1 (IL-1), interleukin-6 (IL-6), and tumor necrosis factor-alpha (TNF-α), which enhance the binding of ubiquitin to target proteins and accelerate protein degradation contributing to muscle contractile dysfunction by inducing the expression of E3 ligases [41].

Currently, the strong evidence regarding the role of sarcopenia and systemic inflammation in the prognosis of esophageal cancer is limited. A prospective cohort study involving 2470 patients with colorectal cancer revealed the positive association between sarcopenia and high NLR levels, and their combination could effectively identify the poorer prognoses of patients with early colorectal cancer [42]. Our study first discovered sarcopenia as an independent prognostic factor for patients with esophageal cancer receiving chemoradiotherapy. Simultaneously, we outlined NLR as a predictive factor for sarcopenia. The outcomes indicate that the combined sarcopenia–NLR model has better predictive power than considering sarcopenia or NLR alone and has been validated as an independent prognostic factor for esophageal cancer patients undergoing chemoradiotherapy. Interventions that affect tumor growth and treatment resistance for patients at higher risk can help improve their quality of life and survival prognosis.

However, our research has particular limitations. First, it is designed as retrospective, and the assessment of sarcopenia was based on imaging to evaluate SMI in patients, in whom the data on muscle function were not provided. Second, as a single-center study with a relatively small sample size, biases may exist that warrant validation in larger multi-center prospective studies. Furthermore, the complex network between tumor inflammation and body composition and its underlying mechanisms still requires further investigation.

## Conclusion

Our study has combined two predictive factors of NLR and sarcopenia across different treatment processes that serve as one independent prognostic for patients with esophageal cancer receiving chemoradiotherapy. This predictive model is characterized by simplicity, reliability, and effective identification of high-risk patients and has certain clinical guiding significance.

### Supplementary Information

Supplementary Material 1.

## Data Availability

The original data supporting the findings of this study are available from the corresponding author upon reasonable request. Corresponding Author: Tao Li, email: litaoxmf@126.com.
