# Supplementary material for: Prognostic value of sarcopenia and inflammatory indices synergy in patients with esophageal squamous cell carcinoma undergoing chemoradiotherapy
Source: BMC Cancer. 2024 Jul 18;24:860. doi: 10.1186/s12885-024-12602-1 (PMC11256500; doi:10.1186/s12885-024-12602-1)

Supplementary figure 1

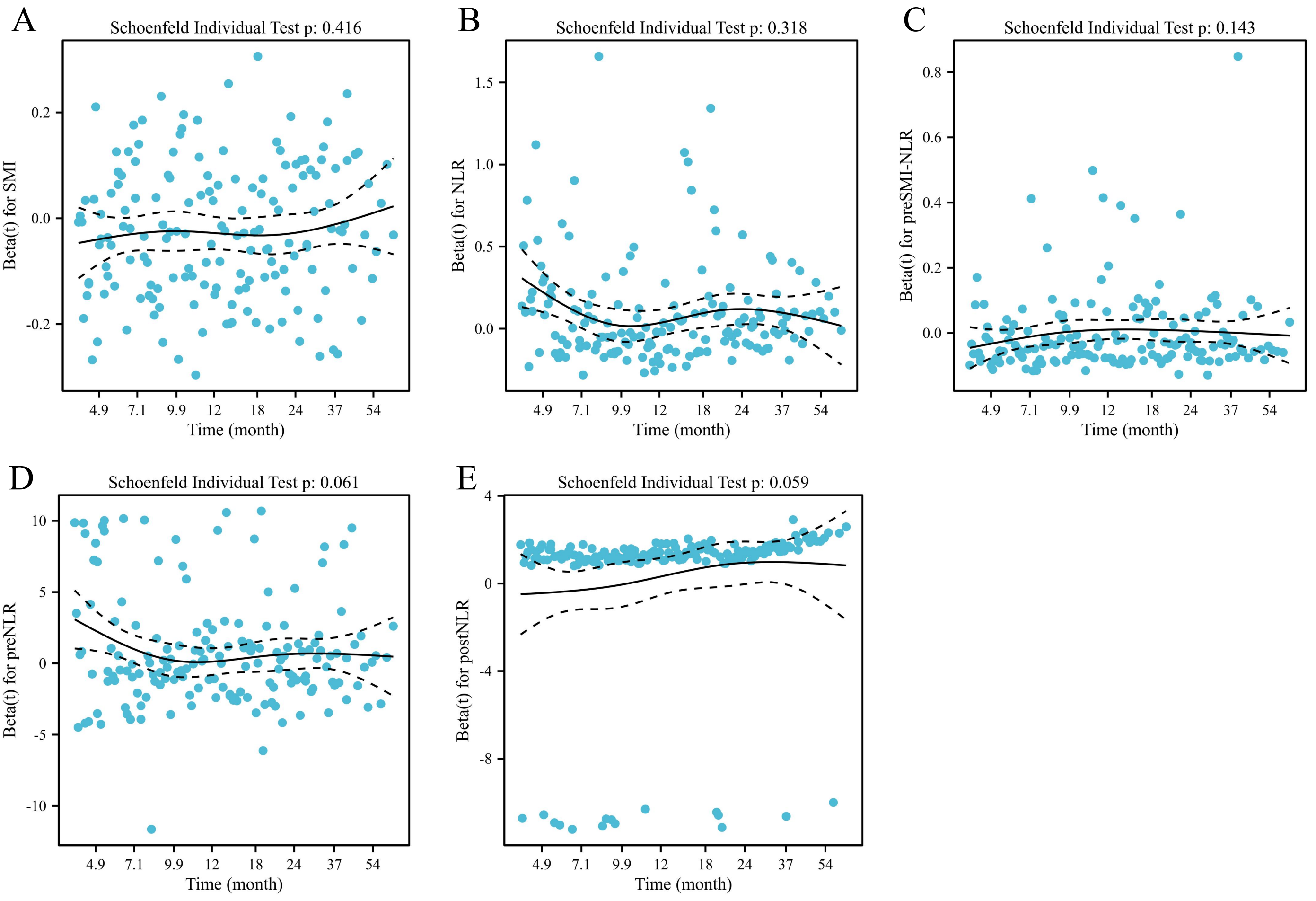

Supplementary table 2

Group	Risk	n.event	Total number of censors	Total censoring ratio	Median survival time	Median survival time confidence interval
-	255	165	90	0.353	24.3	20.33-33.8

Supplementary figure 2

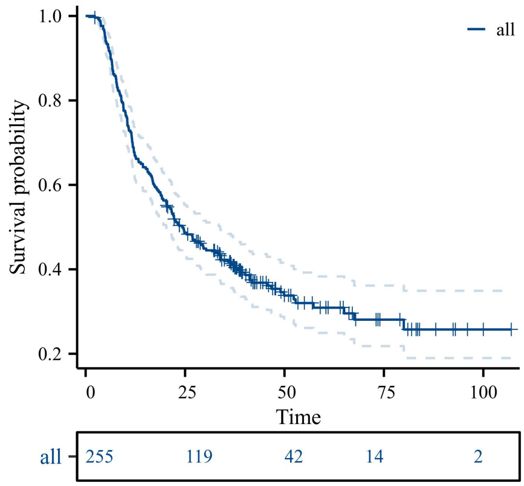

Supplementary figure 3

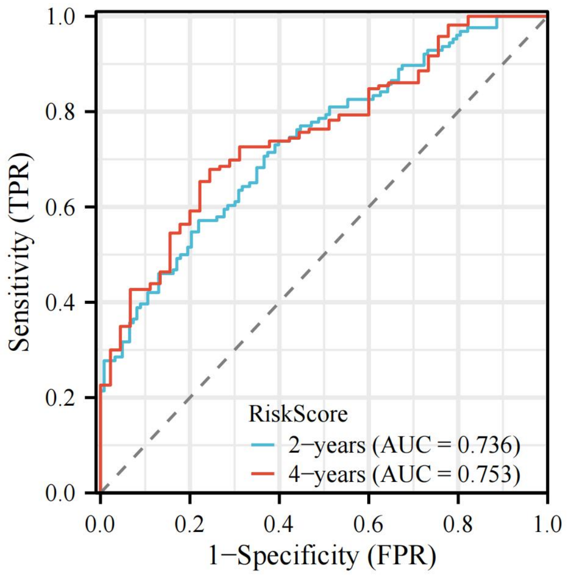

Supplement: Supplementary file 1 — Supplementary Material 1. [file 12885_2024_12602_MOESM1_ESM.pdf]
